# Supplementary material for: Financial burden of cancer in Nepal: Factors associated with annual cost and catastrophic health expenditure
Source: PLoS One. 2025 Sep 3;20(9):e0331321. doi: 10.1371/journal.pone.0331321 (PMC12407443; doi:10.1371/journal.pone.0331321)
Supplement: S1 File — (DOCX) [file pone.0331321.s001.docx]

**Supplementary File**

**Financial burden of cancer in Nepal: Determinants of annual cost and catastrophic health expenditure**

**Authors and affiliation**

Pratik Khanal^1*^, Kjell Arne Johansson^1^, Achyut Raj Pandey^1^, Ravi Kant Mishra^1^, Nirmal Poudel^2^, Sandipa Sharma^2^, Biraj Man Karmacharya^3^, Shiva Raj Adhikari^2,4^, Krishna Kumar Aryal^1^

^1^Bergen Centre for Ethics and Priority Setting in Health (BCEPS), Department of Global Public Health and Primary Care, University of Bergen, Bergen, Norway

^2^Nepal Health Economics Association, Kathmandu, Nepal

^3^Department of Public Health and Community Programs, Kathmandu University School of Medical Sciences, Dhulikhel, Nepal’

^4^Central Department of Economics, Tribhuvan University, Kathmandu, Nepal

**Correspondence:** [**iampratikkhanal@gmail.com**](mailto:iampratikkhanal@gmail.com) **(PK)**

**Supplementary Tables**

S1 Table. Study variables

S2 Table. Utilization of Deprived citizen treatment fund by patients with cancer

S3 Table. Association of socio-demographic and treatment related variables with annual cost of cancer care

S4 Table. Association between independent variables and CHE incidence at 25% threshold

S5 Table. Average cost of cancer care in USD at different time frames

**Supplementary Figures**

S1 Fig. Membership in social health protection schemes (%)

**S1 Table. Study variables**

| **S.N.** | **Variables** | **Categories of variables** |
| --- | --- | --- |
|  | **Patient cost related variables** |  |
| 1 | Direct medical costs | consultation cost, treatment cost, investigation cost, drug cost, other medical costs. |
| 2 | Direct non-medical costs | food cost, accommodation cost, transportation cost, clothing cost, caretaker expenses, other non-medical costs |
| 3 | Indirect costs | productivity loss of patients, productivity loss of caretakers |
| 4 | Cost of cancer care | Sum of direct medical, direct non-medical, and indirect costs |
| 5 | Catastrophic health expenditure | Calculated at 10 and 25 percent threshold of annual household expenditure |
| 6 | Impoverishment | Calculated as the proportion of the population falling below the poverty line after deducting out-of-pocket payments |
| 7 | Sources of finance for cancer care | current income of any household member/s or household income,  own or household members saving, support from relatives, sale of assets,  borrowed or taking loans from individuals and financial institutions, donation/charity from friends and relatives, covered through insurance, subsidy from the NGO or other agencies, subsidies from local government, other sources |
| 8 | Consequences of cancer treatment | cut back on food and other household consumption, cut back on non-food expenditure such as clothing, transport, education, applied for financial assistance, borrowed money from family or friends, reduced medical visits, switched hospital, skipped medicine or recommended care |
|  | **Socio-demographic characteristics** |  |
| 1 | Age | In years, categorized as 20-39 years, 40-59 years, and 60 and above |
| 2 | Gender | female, male |
| 3 | Ethnicity | Hill Brahmin/Chhetri, Janajati, Madheshi, Dalit, Muslim, and others. Dalits and Muslim were grouped as others during analysis. Adapted as per Nepal’s Health Management Information System |
| 4 | Religion | Hindu, Buddhist, others. Others include Christian, Muslim and Kirat |
| 5 | Province | Koshi, Madhesh, Bagmati, Gandaki, Lumbini, Karnali, and Sudurpashchim. For analysis, Karnali and Sudurpaschchim were grouped together. |
| 6 | Residence | Urban, Rural |
| 7 | Education | no formal education, basic education, secondary education and above |
| 8 | Type of family | Nuclear and joint/extended |
| 9 | Family size | up to five, greater than five |
| 10 | Marital status | Currently married, not currently in union (never married, divorced, separated, widowed) |
| 11 | Occupation | not working and did not work in the last 12 months, agriculture, employed (sales and service, professional/technical/managerial, unskilled manual, skilled manual, clerical) and others (including homemaker) |
| 12 | Number of economically active family members | None, one, greater than one |
| 13 | Annual household expenditure | In Nepalese rupees, average household monthly expenditure was multiplied by 12 to obtain annual household expenditure |
| 14 | Wealth quintile | lowest, lower, middle, higher and highest. based on annual household expenditure. |
|  | **Treatment related variables** |  |
| 1 | Type of cancer | Lung, Cervical, Breast, Stomach, and Oesphagus |
| 2 | Duration of diagnosis | In months, categorized as less than six months, 6 months to one year, above one year |
| 3 | Staging of cancer | early stage (stage I and II), advanced stage (stage III and IV), not mentioned |
| 4 | Duration of treatment | in months, categorized as less than six months, 6 months to one year, above one year |
| 5 | Treatment modality | singular, combination |
| 6 | Visited private health facility before coming to the study hospital | yes, no |
| 7 | Presence of other chronic diseases | yes, no |
| 8 | Study site | BP Koirala Memorial Cancer Hospital, Bhaktapur Cancer Hospital |
| 9 | Travelling distance to the study site | less than one hour, 1 hour to 6 hours, greater than 6 hours |
| 10 | Admission to inpatient care last year (past 365 days) | yes, no |
|  | **Social health protection scheme related variables** |  |
| 1 | Member of national health insurance scheme | yes, no |
| 2 | Member of any health protection scheme | yes, no. includes membership in national health insurance, employee provident fund, social security fund, private health insurance |
| 3 | Heard about deprived citizen treatment fund | yes, no |
| 4 | Received treatment subsidy for cancer from at least one sources | yes, no. treatment subsidy includes subsidy received from federal and provincial sources including monthly cash allowance |

**S2 Table. Utilization of Deprived citizen treatment fund by patients with cancer**

| **Characteristics** | **Categories** | **Frequency** | **Percentage** |
| --- | --- | --- | --- |
| Heard about deprived citizen treatment fund (n=353) | Yes | 328 | 92.9 |
| Utilization of subsidy related to cancer (n=328) | Utilized subsidy from the federal government | 288 | 87.8 |
|  | Utilized subsidy from the province government | 59 | 18.0 |
|  | Received monthly allowance from the local government | 57 | 17.4 |
|  | Utilized subsidies from all above three sources | 25 | 7.6 |
|  | Did not utilize any of the subsidy | 37 | 11.3 |

**S3 Table. Association of socio-demographic and treatment related variables with annual cost of cancer care**

| **Characteristics** | **N** | **Annual mean cost in USD** | **SD** | **Mean rank** | **Test statistics** | **p-value** |
| --- | --- | --- | --- | --- | --- | --- |
| **Age (years)** |  |  |  |  |  |  |
| 20-39 | 48 | 3954 | 3704 | 175.98 | 2.615 | 0.270 |
| 40-59 | 158 | 4087 | 3838 | 186.27 |  |  |
| 60 and above | 147 | 3171 | 2010 | 167.37 |  |  |
| **Gender** |  |  |  |  |  |  |
| Male | 96 | 3426 | 2303 | 175.57 | -1.61 | 0.872 |
| Female | 257 | 3785 | 3481 | 177.53 |  |  |
| **Ethnicity** |  |  |  |  |  |  |
| Hill Brahmin/Chhetri | 106 | 4098 | 3468 | 191.55 | 9.209 | 0.027 |
| Madhesi | 42 | 3448 | 3562 | 160.79 |  |  |
| Janajati | 163 | 3385 | 3094 | 206.17 |  |  |
| Others | 42 | 4062 | 2420 |  |  |  |
| **Religion** |  |  |  |  |  |  |
| Hindu | 283 | 3593 | 3267 | 173.77 | 1.935 | 0.380 |
| Buddhist | 47 | 4073 | 3145 | 184.02 |  |  |
| Others | 23 | 4061 | 2496 | 202.43 |  |  |
| **Province** |  |  |  |  |  |  |
| Koshi | 46 | 4273 | 3024 | 200.50 | 10.771 | 0.056 |
| Madhesh | 32 | 3251 | 3904 | 144.22 |  |  |
| Bagmati | 126 | 3881 | 2781 | 189.98 |  |  |
| Gandaki | 55 | 3238 | 3412 | 154.55 |  |  |
| Lumbini | 57 | 3730 | 4134 | 169.25 |  |  |
| Karnali and Sudurpashchim | 37 | 3277 | 1935 | 177.27 |  |  |
| **Residence** |  |  |  |  |  |  |
| Urban | 249 | 3749 | 3459 | 175.84 | -0.331 | 0.741 |
| Rural | 104 | 3540 | 2503 | 179.78 |  |  |
| **Educational qualification** |  |  |  |  |  |  |
| No formal education | 205 | 3370 | 2246 | 175.46 | 9.723 | 0.008 |
| Basic education | 78 | 3460 | 3554 | 154.50 |  |  |
| Secondary education and above | 70 | 4870 | 4648 | 206.59 |  |  |
| **Type of family** |  |  |  |  |  |  |
| Nuclear | 134 | 3678 | 3186 | 177.42 | -0.060 | 0.952 |
| Joint/Extended | 190 | 3693 | 3223 | 176.74 |  |  |
| **Marital status** |  |  |  |  |  |  |
| Married | 300 | 3656 | 3131 | 177.06 | -0.026 | 0.979 |
| Not currently in union | 53 | 3866 | 3621 | 176.66 |  |  |
| **Study site** |  |  |  |  |  |  |
| BPKMCH | 186 | 3260 | 3226 | 158.17 | -3.658 | <.001 |
| BCH | 167 | 4163 | 3122 | 197.97 |  |  |
| **Travel time** |  |  |  |  |  |  |
| Less than one hour | 56 | 3987 | 3856 | 173.68 | 0.487 | 0.784 |
| One hour to six hours | 157 | 3768 | 3091 | 181.24 |  |  |
| More than 6 hours | 140 | 3476 | 3052 | 173.58 |  |  |
| **Family size** |  |  |  |  |  |  |
| Up to 5 | 191 | 3804 | 3561 | 178.21 | -.243 | 0.808 |
| Greater than 5 | 162 | 3550 | 2729 | 175.57 |  |  |
| **Occupation** |  |  |  |  |  |  |
| Not working and did not work in last 12 months | 156 | 4025 | 3461 | 191.01 | 11.947 | 0.008 |
| Employed | 66 | 4117 | 3737 | 188.09 |  |  |
| Agriculture | 76 | 3305 | 2679 | 165.43 |  |  |
| Others | 55 | 2742 | 2028 | 139.95 |  |  |
| **Number of economically active family members** |  |  |  |  |  |  |
| None | 18 | 5234 | 6507 | 204.17 | 1.821 | 0.402 |
| One | 174 | 3452 | 2465 | 171.84 |  |  |
| Two or more | 161 | 3769 | 3345 | 179.54 |  |  |
| **Wealth quintile** |  |  |  |  |  |  |
| Lowest | 72 | 2834 | 1670 | 154.13 | 14.536 | 0.006 |
| Lower | 82 | 3702 | 3639 | 174.23 |  |  |
| Middle | 46 | 3034 | 2449 | 150.70 |  |  |
| Higher | 82 | 3789 | 2678 | 186.77 |  |  |
| Highest | 71 | 4841 | 4352 | 209.15 |  |  |
| **Member of NHIP** |  |  |  |  |  |  |
| No | 160 | 3452 | 3675 | 161.16 | -2.655 | .008 |
| Yes | 193 | 3882 | 2749 | 190.13 |  |  |
| **Member of any health protection scheme** |  |  |  |  |  |  |
| None | 146 | 3307 | 3120 | 159.55 | -2.697 | .007 |
| At least one | 207 | 3956 | 3243 | 189.30 |  |  |
| **Received chronic disease-related subsidy from at least one sources** |  |  |  |  |  |  |
| None | 37 | 2453 | 1726 | 128.41 | -3.062 | .002 |
| At least one source | 316 | 3832 | 3307 | 182.69 |  |  |
| **Type of cancer** |  |  |  |  |  |  |
| Lungs | 89 | 3810 | 3262 | 181.62 | 5.611 | 0.230 |
| Breast | 82 | 3819 | 3290 | 181.68 |  |  |
| Cervical | 92 | 3275 | 2475 | 164.67 |  |  |
| Stomach | 57 | 4357 | 4247 | 196.70 |  |  |
| Oesophagus | 33 | 3021 | 2345 | 153.24 |  |  |
| **Duration of diagnosis** |  |  |  |  |  |  |
| < 6 months | 199 | 2604 | 1771 | 137.61 | 68.497 | <.001 |
| 6 months to 1 year | 95 | 5162 | 4215 | 232.67 |  |  |
| > one year | 59 | 4965 | 3684 | 220.20 |  |  |
| **Cancer staging** |  |  |  |  |  |  |
| Early stage and not mentioned | 189 | 3190 | 2747 | 159.80 | -3.400 | <.001 |
| Advanced stage | 164 | 4260 | 3586 | 196.82 |  |  |
| **Duration of treatment** |  |  |  |  |  |  |
| Less than 6 months | 211 | 2619 | 1732 | 139.32 | 73.381 | <.001 |
| 6 months to 1 year | 85 | 5451 | 4323 | 242.59 |  |  |
| Above one year | 57 | 5012 | 3803 | 218.67 |  |  |
| **Treatment modality** |  |  |  |  |  |  |
| Singular | 239 | 3034 | 2642 | 153.69 | -6.214 | <.001 |
| Combination | 114 | 5058 | 3807 | 225.87 |  |  |
| **Visited private health facility** |  |  |  |  |  |  |
| No | 135 | 3340 | 2346 | 167.39 | -1.393 | .164 |
| Yes | 218 | 3903 | 3625 | 182.95 |  |  |
| **Presence of other chronic diseases** |  |  |  |  |  |  |
| No | 235 | 3513 | 3082 | 170.92 | -1.579 | .114 |
| Yes | 118 | 4034 | 3423 | 189.10 |  |  |
| **Admission to inpatient care** |  |  |  |  |  |  |
| No | 110 | 2684 | 1781 | 142.51 | -4.273 | <0.001 |
| Yes | 243 | 4141 | 3584 | 192.61 |  |  |

**S4 table. Association between independent variables and CHE incidence at 25% threshold**

| **Characteristics** | **No CHE** | **CHE** | **Chi-square value** | **p-value** |
| --- | --- | --- | --- | --- |
| **Age (years)** |  |  |  |  |
| 20-39 | 7 (14.6) | 41 (85.4) | 0.917 | 0.632 |
| 40-59 | 23 (14.6) | 135 (85.4) |  |  |
| 60 and above | 27 (18.4) | 120 (81.6) |  |  |
| **Gender** |  |  |  |  |
| Male | 14 (14.6) | 82 (85.4) | 0.238 | 0.626 |
| Female | 43 (16.7) | 214 (83.3) |  |  |
| **Ethnicity** |  |  |  |  |
| Hill Brahmin/Chhetri | 13 (12.3) | 93 (87.7) | 4.399 | 0.221 |
| Madhesi | 9 (21.4) | 33 (78.6) |  |  |
| Janajati | 31 (19.0) | 132 (81.0) |  |  |
| Others | 4 (9.5) | 38 (90.5) |  |  |
| **Religion** |  |  |  |  |
| Hindu | 45 (15.9) | 238 (84.1) | 0.065 | 0.968 |
| Buddhist | 8 (17.0) | 39 (83.0) |  |  |
| Others | 4 (17.4) | 19 (82.6) |  |  |
| **Province** |  |  |  |  |
| Koshi | 6 (13.0) | 40 (87.0) | 5.801 | 0.326 |
| Madhesh | 7 (21.9) | 25 (78.1) |  |  |
| Bagmati | 21 (16.7) | 105 (83.3) |  |  |
| Gandaki | 13 (23.6) | 42 (76.4) |  |  |
| Lumbini | 7 (12.3) | 50 (87.7) |  |  |
| Karnali and Sudurpashchim | 3 (8.1) | 34 (91.9) |  |  |
| **Residence** |  |  |  |  |
| Urban | 44 (17.7) | 205 (82.3) | 1.449 | 0.229 |
| Rural | 13 (12.5) | 91 (87.5) |  |  |
| **Educational qualification** |  |  |  |  |
| No formal education | 34 (16.6) | 171 (83.4) | 3.081 | 0.214 |
| Basic education | 16 (20.5) | 62 (79.5) |  |  |
| Secondary education and above | 7 (10.0) | 63 (90.0) |  |  |
| **Type of family** |  |  |  |  |
| Nuclear | 12 (9.0) | 122 (91.0) | 8.251 | 0.004 |
| Joint/Extended | 45 (20.5) | 174 (79.5) |  |  |
| **Marital status** |  |  |  |  |
| Married | 46 (15.3) | 254 (84.7) | 0.978 | 0.323 |
| Not currently in union | 11 (20.8) | 42 (79.2) |  |  |
| **Study site** |  |  |  |  |
| BPKMCH | 30 (16.1) | 156 (83.9) | 0.001 | 0.992 |
| BCH | 27 (16.2) | 140 (83.8) |  |  |
| **Travel time** |  |  |  |  |
| Less than one hour | 13 (23.2) | 43 (76.8) | 2.757 | 0.252 |
| One hour to six hours | 25 (15.9) | 132 (84.1) |  |  |
| More than 6 hours | 19 (13.6) | 121 (86.4) |  |  |
| **Family size** |  |  |  |  |
| Up to 5 | 19 (9.9) | 172 (90.1) | 11.814 | <0.001 |
| Greater than 5 | 38 (23.5) | 124 (76.5) |  |  |
| **Occupation** |  |  |  |  |
| Not working and did not work in last 12 months | 23 (14.7) | 133 (85.3) | 1.607 | 0.658 |
| Employed | 13 (19.7) | 53 (80.3) |  |  |
| Agriculture | 14 (18.4) | 62 (81.6) |  |  |
| Others | 7 (12.7) | 48 (87.3) |  |  |
| **Number of economically active family members** |  |  |  |  |
| None or one | 19 (9.9) | 173 (90.1) | 12.151 | <0.001 |
| Two or more | 38 (23.6) | 123 (76.4) |  |  |
| **Wealth quintile** |  |  |  |  |
| Lowest | 1 (1.4) | 71 (98.6) | 49.805 | <0.001 |
| Lower | 6 (7.3) | 76 (92.7) |  |  |
| Middle | 10 (21.7) | 36 (78.3) |  |  |
| Higher | 11 (13.4) | 71 (86.6) |  |  |
| Highest | 29 (40.8) | 42 (59.2) |  |  |
| **Member of NHIP** |  |  |  |  |
| No | 28 (17.5) | 132 (82.5) | 0.395 | 0.529 |
| Yes | 29 (15.0) | 164 (85.0) |  |  |
| **Member of any health protection scheme** |  |  |  |  |
| None | 23 (15.8) | 123 (84.2) | 0.029 | 0.866 |
| At least one | 34 (16.4) | 173 (83.6) |  |  |
| **Received chronic disease-related subsidy from at least one sources** |  |  |  |  |
| None | 8 (21.6) | 29 (78.4) | 0.915 | 0.339 |
| At least one source | 49 (15.5) | 267 (84.5) |  |  |
| **Type of cancer** |  |  |  |  |
| Lungs | 14 (15.7) | 75 (84.3) | 0.812 | 0.937 |
| Breast | 13 (15.9) | 69 (84.1) |  |  |
| Cervical | 13 (14.1) | 79 (85.9) |  |  |
| Stomach | 11 (19.3) | 46 (80.7) |  |  |
| Oesophagus | 6 (18.2) | 27 (81.8) |  |  |
| **Duration of diagnosis** |  |  |  |  |
| < 6 months | 35 (17.6) | 164 (82.4) | 9.462 | 0.009 |
| 6 months to 1 year | 7 (7.4) | 88 (92.6) |  |  |
| > one year | 15 (25.4) | 44 (74.6) |  |  |
| **Cancer staging** |  |  |  |  |
| Early stage and not mentioned | 31 (16.4) | 158 (83.6) | 0.020 | 0.889 |
| Advanced stage | 26 (15.9) | 138 (84.1) |  |  |
| **Duration of treatment** |  |  |  |  |
| Less than 6 months | 40 (19.0) | 171 (81.0) | 17.529 | <0.001 |
| 6 months to 1 year | 2 (2.4) | 83 (97.6) |  |  |
| Above one year | 15 (26.3) | 42 (73.7) |  |  |
| **Treatment modality** |  |  |  |  |
| Singular | 42 (17.6) | 197 (82.4) | 1.111 | 0.292 |
| Combination | 15 (13.2) | 99 (86.8) |  |  |
| **Visited private health facility** |  |  |  |  |
| No | 25 (18.5) | 110 (81.5) | 0.908 | 0.341 |
| Yes | 32 (14.7) | 186 (85.3) |  |  |
| **Presence of other chronic diseases** |  |  |  |  |
| No | 42 (17.9) | 193 (82.1) | 1.545 | 0.214 |
| Yes | 15 (12.7) | 103 (87.3) |  |  |
| **Admission to inpatient care** |  |  |  |  |
| No | 27 (24.5) | 83 (75.5) | 8.324 | 0.004 |
| Yes | 30 (12.3) | 213 (87.7) |  |  |

**S5 Table. Average cost of cancer care in USD at different time frames**

| **Cost of cancer at different time frames (USD)** | **Direct medical cost**  Mean (SD) | **Direct non-medical cost**  Mean (SD**)** | **Total direct cost**  Mean (SD) |
| --- | --- | --- | --- |
| Cost during this visit (outpatient, n=195) | 138.4 (188.1) | 81.4 (150.7) | 219.78 (276.4) |
| Cost during this visit (inpatient, n=158) | 436.0 (592.8) | 128.0 (166.8) | 564.02 (698.8) |
| Cost in last one year (includes cost of this visit) | 1982.7 (2226.6) | 581.9 (827.8) | 2564.66 (2694.1) |
| Cost from the time of cancer diagnosis to the survey period (includes cost of this visit) | 2568.6 (4719.0) | 741.6 (1210.8) | 3310.17 (5603.1) |

**Financial protection related characteristics**

Among study participants, 54.7% were members of the NHIP while 4% of the members had private insurance. Similarly, 6.2% and 2.3% of the participants were members of the state-run social security fund (SSF) and employee provident fund (EPF), respectively. The SSF provides health insurance for the private sector while the EPF provides health insurance for public sector employees. Two in five participants were not members of any of the social health protection schemes (S1 Fig).

**S1 Fig. Membership in social health protection schemes (%)**
