# Supplementary material for: Financial burden of cancer in Nepal: Factors associated with annual cost and catastrophic health expenditure
Source: PLoS One. 2025 Sep 3;20(9):e0331321. doi: 10.1371/journal.pone.0331321 (PMC12407443; doi:10.1371/journal.pone.0331321)
Supplement: S2 File — (PDF) [file pone.0331321.s002.pdf]

Cancer Costing study (Patient's Perspective)

Patient ID *

Date of interview (अन्तरवार्ता मिति)

yyyy-mm-dd hh:mm

Name of interviewer (इन्टरभ्यू गर्ने व्यक्तिको नाम) *

Place of interview (स्थान) *

- Outpatient department (ओ.पी.डी)
- Inpatient ward (आई.पी.डी)
- Emergency department
- Within the hospital premises (अस्पताल परिसर)
- Others (specify) अन्य (उल्लेख गर्नुहोस)

Name of the hospital (अस्पतालको नाम) *

- BP Koirala Memorial Cancer Hospital, Bharatpur (वि . पी कोइराला मेमोरियल क्यान्सर अस्पताल , भरतपुर)
- Bhaktapur Cancer Hospital (भक्तपुरक्यान्सर अस्पताल)

Travel

Means of transportation *

- Public vehicle
- Private vehicle
- Own vehicle

Travelling distance to hospital from home *

(In hour and minutes)

Section I: Socio-demographic information

Age (तपाई कति वर्ष पुरा हुनुभयो ?) *

Ask their date of birth to verify

in years

Gender (तपाईको लैङ्गिक पहिचान?) *

- Male(पुरुष)
- Female (महिला)
- Others

Place of residence (क्षेत्र) *

- Urban (Metropolitan, sub-metropolitan, Municipality) महानगरपालिका, उप-महानगरपालिका, नगरपालिका
- Rural Municipality (गाउँपालिका)

province

- ◯ Koshi
- ◯ Madhesh
- ◯ Bagmati
- ◯ Gandaki
- ◯ Lumbini
- ◯ Karnali
- ◯ Sudurpaschim

district

Marital status (वैवाहिक स्थिति) *

- ◯ Never in union (अविवाहित)
- ◯ Married (विवाहित)
- ◯ Living with partner (लिभ इन टुगेदर)
- ◯ Widowed (एकल महिला)
- ◯ Divorced (डिभोर्स गरेको)
- ◯ Separated (छुट्टिएर बसेको)

Educational qualification (शैक्षिक योग्यता) *

- ◯ No formal education (औपचारिक शिक्षा नभएको)
- ◯ Lower basic education (1-5) (निम्न आधारभुत शिक्षा १–५)
- ◯ Upper basic education (6-8) (उच्च आधारभुत शिक्षा ६–८)
- ◯ Lower secondary (9-10) (निम्न माध्यामिक ९-१०)
- ◯ Higher secondary (11-12) or certificate level (उच्च माध्यामिक ११-१२)
- ◯ Bachelors (स्नातक)
- ◯ Master's degree and above (स्नातकोत्तर तह वा सो भन्दा माथि)
- ◯ No formal education but can read and write (पढ्न लेख्न सक्ने तर औपचारिक शिक्षा नभएको)

Ethnicity (जातजाति) *

- Hill Brahmin (ब्राह्मण पहाड))
- Hill Chhetri (क्षेत्री पहाड))
- Terai Brahmin/Chhetri ब्राह्मण/ क्षेत्री तराई)
- Other Terai caste (तराई अन्य जाति)
- Hill Dalit (दलित –पहाड)
- Terai Dalit (दलित –तराई)
- Newar (नेवार)
- Hill Janajati (जनजाति –पहाड)
- Terai Janajati (जनजाति –तराई)
- Muslim (मुस्लिम)
- Other (अन्य)

Religion (धर्म) *

- Hindu (हिन्दु)
- Buddhist (बौद्ध)
- Muslim (मुस्लिम)
- Kirat (किरात)
- Christian (क्रिस्चियन)
- Other (अन्य) specify

Type of family (परिवारको किसिम) *

- Nuclear (एकल)
- Joint (संयुत)
- Extended (विस्तारित)

Number of family members (परिवारको सदस्य संख्या) *

....

Number of children in your family (less than 15 years) परिवारमा १५ वर्ष मुनिका सदस्य संख्या *

....

Number of elderly in your family (65 and above) परिवारमा ६५ वर्ष माथिका सदस्य संख्या *

....

Number of family members with chronic conditions (in number starting from 0) परिवारका सदस्यहरूमा अवस्थित दिर्घ स्वास्थ्य संक्रमण भएका संख्या (संख्या शुरू गर्दा ० बाट) *

Excluding cancer patient

Estimated monthly expenses of other family member with chronic diseases दिर्घरोगको अन्य परिवारका सदस्यहरूको मासिक खर्चको अनुमानित (रकम

Number of family members who are economically active (परिवारमा आर्थिक रुपमा सक्रिय सदस्य संख्या) *

Occupation (तपाइको पेशा) *

- ◯ Not working and didn't work in last 12 months (पछिल्लो १२ महिना देखि कुनै पेशामा आबद्ध नभएको)
- ◯ Professional/teaching/managerial (प्रोफेश्नल/टेक्निकल/म्यानेजिरियल)
- ◯ Clerical (क्लेरिक) such as office assistant, data entry, filing documents
- ◯ Sales and service (सेल्स/सर्भिस) such as business, self employed
- ◯ Skilled manual (दक्ष म्यानुअल) such as carpentry, plumber, technician
- ◯ Unskilled manual (अदक्ष म्यानुअल) such as construction labor
- ◯ Agriculture (कृषि)
- ◯ Other (अन्य)

If others, specify

Employment status (तपाईको रोजगारीको अवस्था)

- ◯ Employed full time (पुर्ण समय रोजगार)
- ◯ Employed part time (आंशिक समय रोजगार)
- ◯ On Leave (विदामा वसेको)
- ◯ Retired because of cancer (left job due to cancer) (क्यान्सरका कारण सेवानिवृत्त)
- ◯ Retired due to age (उमेरका कारण सेवानिवृत्त)
- ◯ Not working outside home (घर बहिर काम नगरेको)
- ◯ Student (विद्यार्थी)
- ◯ Homemaker (गृहणी)

Monthly income of the individual (if currently employed or previously employed left due to cancer within last 12 months) पछिल्लो 12 महिना भित्र तपाईको मासिक आम्दानी कति छ/थियो ?

In NRS

Household monthly income (average) (तपाईको परिवारको औसत मासिक आम्दानी ?) *

In NRS

Household monthly expenditure (average) (तपाईको परिवारको औसत मासिक आर्थिक खर्च ?) *

In NRS

Are you insured under National Health Insurance Program of Government? (के तपाई राष्ट्रिय स्वास्थ्य विमा कार्यक्रममा आबद्ध हुनुहुन्छ ?) *

- No (छैन)
- Yes (छु)

» Insurance

Are you a member of any of the following? (तपाईं कुनै पनि निम्नलिखितको सदस्य हुनुहुन्छ?)

Employment provident fund (कर्मचारी सञ्चयकोष)

- No
- Yes

Social Security Fund (सामाजिक सुरक्षा कोष)

- No
- Yes

Citizen Investment Trust (नागरिक लगानी कोष)

- No
- Yes

Private insurance (निजी विमा कम्पनीको विमा योजना)

- No
- Yes

Any other scheme that fully or partly provide support in treatment (कुनै अरु योजना जसले पूर्ण वा आंशिक रूपमा उपचारमा सहायता प्रदान गर्दछ?)

- No
- Yes

If yes, specify

If private insurance, specify the amount of the coverage (यदि निजी बीमा भए, वरेजको रकम स्पष्ट गर्नुहोस्

» Bipanna Nagarik Kosh

Have you heard about Bipanna Nagarik Kosh whereby government provides subsidy of 100,000 in the treatment of cancer patients?(के तपाईंले "बिपन्न नागरिक कोष" बारेमा सुनेका छिन्, जसमा सरकारले क्यान्सर रोगीहरूको उपचारमा १००,००० को अनुदान प्रदान गर्दछ?) *

- No (छैन)
- Yes (छु)
- Don't know

Have you utilized Bipanna Nagarik Kosh Scheme where patient get treatment subsidy from the hospital? (के तपाईले विपन्न नागरिक उपचार कोषको अनुदान लिनु भएको छ ?)

- No (छैन)
- Yes, have utilized subsidy from the federal government (छ संघीय सरकारबाट अनुदान प्रयोग गरेका छौं)
- Yes, have utilized subsidy from the province government (प्रदेश सरकारबाट अनुदान प्रयोग गरेका छु)
- Yes, have received additional monthly allowance (मासिक अतिरिक्त भत्ता प्राप्त गरेको छ।)
- Planning to utilize (उपयोग गर्ने योजना)
- Not necessary (आवश्यक छैन)
- Don't know (थाहा छैन)

Section II: Disease description

» Organs affected

Organs affected (Cancer Site) क्यान्सर प्रभावित अंग ? *

- [ ] Lungs (फोक्सो)
- [ ] Breast (स्तन)
- [ ] Cervical (पाठेघरको मुख)
- [ ] Stomach (पेट)
- [ ] Oesophageal (खाद्यनली)
- [ ] Others (अन्य)

If others, mention अन्य (खुलाउनुहोस)

Check the patient booklet or ask provider

Duration of diagnosis (क्यानसर पहिचान भएको समय ?) (In months) *

Confirm after asking year and month of diagnosis

Months

Treatment stage at the time of diagnosis (क्यान्सरको अवस्था/स्टेज ?) *

- ○ Stage 0 (स्टेज ०)
- ○ Stage I (पहिलो स्टेज)
- ○ Stage II (दोस्रो स्टेज)
- ○ Stage III (तेस्रो स्टेज)
- ○ Stage IV (चौथो स्टेज)
- ○ Not mentioned (पहिचान हुन बाकी)

Duration of being on treatment (उपचारको अवधि) *

In months

Months

For the current treatment, have you visited another health facility before coming to the study hospital? (तपाई यो अस्पतालमा आउनु अघि यस उपचारका लागि अन्य अस्पताल धाउनु भएको थियो ?) *

- No (थिएन)
- Yes (थिए)

How many health facilities have you visited for the treatment of your existing health condition? (तपाईंले आफ्नो वर्तमान स्वास्थ्य स्थितिको उपचारको लागि कति स्वास्थ्य सेवा सुविधामा भ्रमण गरेका छिन्?) *

What type of health facilities have you visited for the treatment of your existing health condition? (तपाईको यस स्वास्थ्य अवस्थाको उपचारको लागि तपाईले कुन प्रकारका स्वास्थ्य संस्थाहरू जानुभएको छ ?)

- Public Hospital (सार्वजनिक अस्पताल)
- Private Hospital (निजी अस्पताल)
- NGO/Cooperative run hospitals (एनजिओ/सहकारी चलिरहेका अस्पतालहरू)
- Pharmacies/Clinics/Polyclinics (फार्मेसी/क्लिनिक/पोलीक्लिनिकहरू)
- Primary health centers and health posts (थमिक स्वास्थ्य केन्द्र र स्वास्थ्य पोस्टहरू)
- Others (Specify) (अन्य)

» Service utilization

Number of outpatient consultations in last one year (तपाईले विगत १ वर्षमा ओ.पी.डी सेवा कति पटक लिनुभयो ?) *

Number of inpatient admissions in last one year (तपाई विगत १ वर्षमा कति पटक अस्पताल भर्ना हुनुभयो ?) *

Number of days hospitalized in last one year (तपाई विगत १ वर्षमा कति दिन अस्पताल भर्ना हुनुभयो ?) *

How many caregivers are involved in taking care of your health on daily basis? (तपाईको स्वास्थ्य उपचारमा दैनिक रुपमा कति हेरचाहकर्ताहरू संलग्न छन्?) *

Which of the following treatments are you currently taking? (दिइएका मध्य तपाईले अहिले कुन उपचार लिइराख्नु भएको छ ?) Multiple choice *

- [ ] Surgery (सर्जरी)
- [ ] Radiation therapy (रेडियसन थेरापी)
- [ ] Chemotherapy (किमो थेरापी)
- [ ] Hormone therapy (हर्मोन थेरापी)
- [ ] Immunotherapy (ईम्युनो थेरापी)
- [ ] Palliative therapy (प्यालिएटिभ थेरापी)
- [ ] Treatment not started (उपचार सुरु भएको छैन)
- [ ] Others (अन्य)

If others, mention अन्य (उल्लेख गर्नुहोस) *

» Service

For which service you are currently in the hospital? *

- ○ Surgery
- ○ Radiation therapy
- ○ Chemotherapy
- ○ Hormone therapy
- ○ Immunotherapy
- ○ Palliative therapy
- ○ Consultation
- ○ Management of complications
- ○ Others

If Others, specify (अन्य उल्लेख गर्नुहोस)

» Comorbidities

Do you have any other chronic disease other than cancer? (के तपाईलाई क्यान्सर बाहेक अन्य कुनै दिर्घ रोगका समस्या छन ?) *

- No (छैन)
- Yes (छ)

If yes, specify (यदि छन भने उल्लेख गर्नुहोस) *

Section III: Direct medical and non-medical cost

Details of health care expenditure (recent visit) (स्वास्थ्य सेवा खर्चको विवरण (हालको भ्रमण))

For patients from outpatient capture the cost of last 7 days

Date of visit *

For inpatient, patient might have been admitted few days before and for outpatient, patient might be in follow up visit or have arrived for collecting reports, you may have to record date that the patient arrived in OPD.

yyyy-mm-dd

Length of in-patient days if hospitalized (अस्पतालमा अस्थायी रूपमा रहेको दिनको अवधि) *

Probable days after which the patient will be discharged if hospitalized (अस्पताल भर्ना भएको कति दिन पछि discharge हुनुभयो *

Verify with provider

» Direct medical cost (recent visit)

For current visit, how much did you spend on each of the following items during the visit (outpatient/inpatient)? हालको भ्रमणमा तपाईंले प्रत्येक वस्तुमा कति खर्च गर्नुभयो

Ticket/Consultation *

Hospital/Bed/Cabin/Fee *

Lab, biopsy/histopathological investigation *

Radiological or Imaging investigations *

Surgery *

Radiation therapy *

Chemotherapy *

Palliative therapy *

Drugs and medical supplies *

Others (if any)

» Direct non-medical costs (recent visit)

For this current visit, how much did you spend on each of the following items during the visit (outpatient/inpatient)? यो हालको भ्रमणको दौरान तपाईंले प्रत्येक निम्नलिखित वस्तुमा कति खर्च गर्नुभयो

Food खाना *

Accommodation (outside hospital setting) of patient बसोबास *

Transport यातायात *

Clothing पोशाक *

Additional expenses for caregiver सहायकको लागि अतिरिक्त खर्च *

Others (if any)

» Direct medical cost in last one year excluding cost of recent visit

Ticket/Consultation *

Hospital/Bed/Cabin/Fee (inpatient) *

Lab investigation including biopsy and histopathological investigation *

Radiological or Imaging investigations *

Surgery *

Radiation therapy *

Chemotherapy *

Palliative therapy *

Drugs and medical supplies *

Others (if any)

» Direct non-medical cost in last one year excluding cost of recent visit

How much did you spend on each of the following items during the visit in last one year (outpatient/inpatient)? बिगत एक बर्षमा प्रत्येक वस्तुमा कति खर्च गर्नुभयो

Food (खाना) *

Accommodation (बसोबास) *

Transport (यातायात) *

Clothing (पोशाक) *

Additional expenses for caregiver (सहायकको लागि अतिरिक्त खर्च) *

Others (if any)

» Total cost from diagnosis till now

From the time of diagnosis to now, how much did you spend on each of the following categories? निदान भएको बेला देखि हालसम्म, तपाईंले प्रत्येक निम्नलिखित वर्गमा कति खर्च गरेका छिन्?

Ask for best possible estimate excluding the cost of recent visits

Direct medical cost *

Direct non-medical cost *

What sources of finance did you use to cover those expenses? (Multiple choice) खर्चहरूमा तपाईंले कुन-कुन वित्तीय स्रोतहरू प्रयोग गरेका छन्

Probe

- [ ] Current income of any household member/s or household income घरका सदस्यहरू वा घरको आम्दानी
- [ ] Own or household member saving आफ्नो वा घरका सदस्यहरूको बचत
- [ ] Support from other members of the following सहायता
- [ ] Sale of assets (land, home, property, livestock, jwellery) or pawning assets (dharauti) सम्पत्तिको बिक्रि
- [ ] Borrowed or taking loans from individuals and financial institutions व्यक्तिहरू र वित्तीय संस्थाबाट कर्जा लिनु वा कर्जा लिनु
- [ ] Donation/charity from friends and relatives मित्रहरू र सम्पर्क सदस्यहरूबाट दान/चारिटी
- [ ] Covered through insurance बीमाबाट कवर गरिएको
- [ ] Subsidy from the NGO or other agencies संगठन वा अन्य एजेन्सीबाट उपनिधि
- [ ] Subsidy from local government
- [ ] Other sources
- [ ] None

If others, specify *

If covered through private insurance or by any other means outside household income, how much money was reimbursed in last one year? प्राइभेट बिमा वा घरको आम्दानी बाहिरको कुनै अन्य तरिकाबाट कवर भएको भए, गत एक वर्षमा कति पैसा प्रतिपूर्ति भएको?

As a result of cancer treatment, have you? उपचारको परिणामस्वरूप, के तपाईंले?

- [ ] cut back on food and other household consumption (खाद्यान्न तथा अन्य घरायसी उपभोग्य वस्तुको उपभोगमा)
- [ ] cut back on non-food expenditure such as clothing, transport, education (गैर खाद्यान्न वस्तु जस्तै लत्ताकपडा, यातायात, शिक्षा आदिको उपभोगमा)
- [ ] applied for financial assistance वित्तीय सहायता आवेदन गरेका छिन्?
- [ ] borrowed money from family or friends (परिवार वा साथीहरुबाट लिएको ऋण तिर्न)
- [ ] reduced medical visits (medical भ्रमणहरूमा कमी गरेका छिन्)
- [ ] switched hospital अस्पताल परिवर्तन गरेका
- [ ] skipped medicine or recommended care औषधि वा सिफारिस गरिएको सेवाहरू छोडेका

Section IV: Indirect cost

» Productivity loss

How many days of work did you miss in last month? (For employed) पछिल्लो महिनामा तपाईले कति दिन काम मा जान छोड्नुभयो ? (रोजगार व्यक्तिका लागि)

How many days of work did you miss in last year?(For employed) विगत एक वर्षमा तपाईले कति दिन काममा जान छोड्नुभयो ? (रोजगार व्यक्तिका लागि)

» Loss in income

Did you suffer any loss in income because of missing work days in last one year? गत एक वर्षमा काम नगर्ने दिनहरूको कारण के तपाईंको आयमा कुनै नुकसान भयो? *

- No
- Yes

If yes, what is the estimated amount of income you lost in last one year गत एक वर्षमा तपाईंले कति आय गुमाउनु भएको

In last one year

How many days of usual work were you unable to do in last one month? (For unemployed including housemaker, elderly or retiree) तपाईले पछिल्लो एक महिनामा घरका अन्य व्यक्तिहरु (गृहणी, वृद्ध तथा सेवानिवृत्त व्यक्ति) लाई हेरचाहको काम गर्न सक्नुभएन ?(रोजगार नभएका व्यक्तिका लागि)

How many days of care work were you unable to do in last one year?(For unemployed including housemaker, elderly or retiree) तपाईले विगत एक वर्षमा घरका अन्य व्यक्तिहरु (गृहणी, वृद्ध तथा सेवानिवृत्त व्यक्ति) लाई हेरचाहको काम गर्न सक्नुभएन ?(रोजगार नभएका व्यक्तिका लागि)

Caregiver relation to the patient (तपाई र तपाईको हेरचाह गर्ने व्यक्तिको सम्बन्ध ?)

- Parent (अभिभावक)
- Children (छोरा/छोरी)
- Sibling (दाजुभाइ/दिदीबहिनी)
- Spouse (पति/पत्नी)
- Other family member (अन्य परिवार सदस्य)
- Friend (साथी)
- Others (अन्य)

Others specify अन्य कोही (उल्लेख गर्नुहोस)

Caregiver gender (तपाईलाई हेरचाह गर्ने व्यक्तिको लैङ्गिक पहिचान ?)

- ◯ Male (पुरुष)
- ◯ Female (महिला)

Caregiver age (completed years) तपाईलाई हेरचाह गर्ने व्यक्तिको उमेर ? (पुरा वर्षमा)

Specify for each caregiver how much time they spent while taking care of the patient or seeking care for illness in last one year? (तपाईलाई हेरचाह गर्ने प्रत्येक व्यक्तिहरुले विगत एक वर्षमा तपाईको हेरचाह गर्दा वा हेरचाह गर्न खोज्दा कति समय बिताउनुभयो ?)

In days

in days

Specify for each caregiver how much time they spent while taking care of the patient in usual day? तपाईलाई हेरचाह गर्ने प्रत्येक व्यक्तिहरुले तपाईको हेरचाह गर्दा वा हेरचाह गर्न खोज्दा एक दिनमा कति समय बिताउनुहुन्छ?

Caretaker 1 (time spent in hours in a day)

Caretaker2

in hour

Caretaker3

in hour

What is your current occupation? (हेरचाह कर्ताको हालको पेशा ?)

Ask caretaker

- ◯ Not working and didn't work in last 12 months (पछिल्लो १२ महिना देखि कुनै पेशामा आबद्ध नभएको)
- ◯ Professional/technical/managerial (प्रोफेश्नल/टेक्निकल/म्यानेजिरियल)
- ◯ Clerical (क्लेरिक)
- ◯ Sales and service (सेल्स/सर्भिस)
- ◯ Skilled manual (स्किल्ड म्यानुअल)
- ◯ Unskilled manual (अनस्किल्ड म्यानुअल)
- ◯ Agriculture (कृषि)
- ◯ Others (अन्य)

If other, specify

How much is your monthly income (for care givers with paid jobs) हेरचाह गर्ने व्यक्तिको मासिक आम्दानी ? (आर्थिक रुपमा सक्रिय हेरचाहकर्ताका लागि)

in rupees

What would you have done with the time if you did not have to take care of the patient? (multiple answers are possible) यदि तपाईले क्यान्सर विरामीलाई हेरचाह गर्नु नपर्ने भको भए तपाई के गर्नुहुन्थ्यो होला ? (हेरचाह गर्ने व्यक्तिलाई, बहुउत्तर प्रश्न)

- ☐ Work (paid) अर्थ (पैसा) आर्जनको काम गर्थे
- ☐ Work (home, non paid) घरायसी काम गर्थे
- ☐ Leisure (फुर्सदमै हुन्थे)
- ☐ Spend with family and friend (परिवार तथा साथीभाइसँग समय विताउथे)
- ☐ Other (Specify) अन्य केही

If others, specify
