# Supplementary material for: Financial burden of cancer in Nepal: Factors associated with annual cost and catastrophic health expenditure
Source: PLoS One. 2025 Sep 3;20(9):e0331321. doi: 10.1371/journal.pone.0331321 (PMC12407443; doi:10.1371/journal.pone.0331321)
Supplement: Attachment — Submitted filename: Response letter_20250711.docx [file pone.0331321.s005.docx]

**11 July 2025**

Editor

Plos One

**Subject: Response to the editorial and reviewer’s comments for the manuscript reference number: PONE-D-25-00853R1**

**Dear Editor,**

First, we would like to thank you for providing us with an opportunity to submit the revised version of our manuscript. We acknowledge the feedback received on the manuscript and have responded to all the comment raised by the academic editor and reviewers. We also have attached a marked-up copy of our manuscript and a clean version of the manuscript. Likewise, we also confirm that figure submitted meet journal requirements as it was developed using Preflight Analysis and Conversion Engine (PACE) digital diagnostic tool.

**Response to reviewer’s comments**

**Reviewer #2:**

Dear Authors, thank you for addressing my comments. Great Job! I hope those were helpful. I have minor comments in the revised version. I hope this will strengthen your manuscript.

**1. Abstract section:**

**- Please add sampling technique in the abstract so that readers can understand your study's generalizability.**

Response: Thank you for the feedback. We now have added sampling technique in the abstract. ‘Face-to-face interviews were conducted with 387 patients undergoing active treatment for lung, breast, cervical, stomach, and oesophagus cancers, recruited using a purposive-consecutive sampling strategy.’

Further, we have expanded sampling techniques to the main body of the manuscript as well.

‘A purposive- consecutive sampling strategy was used to select study participants: five high-burden cancers were purposively selected, and all eligible study patients attending outpatient and inpatient departments of the hospital during the study duration were consecutively recruited until the target sample size was reached’.

**2. You have used the phrase "due to" from abstract to the full text of the manuscript, which infer causality. The phrase “impoverishment due to cancer” implies a direct causal relationship between cancer and impoverishment. The observed association between cancer and impoverishment may be influenced by other unmeasured factors—such as pre-existing economic vulnerability, household income shocks, or healthcare-seeking behaviors—that the study has not accounted for. I recommend replacing “due to” with more appropriate, non-causal language such as “associated with”, “linked to”. Please apply this recommendation throughout the manuscript.**

Response: Thank you for this insightful comment. We now have replaced phrases that imply a causal relationship with a more appropriate non-causal language to reflect the observational nature of our study findings. We have applied the changes throughout the manuscript.

**3. Please add one more limitation for the variable selection in the model. You have selected variables based on the p-value of <0.02 in bivariate analysis. In health economics or health services research, model building should ideally be theory-driven (conceptual framework) rather than data driven. Therefore, I would recommend to add one more limitation.**

Response: Thank you for the feedback. We have added this as one of our study limitations. ‘We also acknowledge that our strategy for variable selection in the regression model was data-driven, which may limit the inclusion of theoretically driven factors.’

Thank you once again for your time and effort in providing constructive feedback on our manuscript.

Regards

**Pratik Khanal**, on behalf of all co-authors
